# A case of tracheal stenosis due to anaplastic thyroid carcinoma treated using the stent‐in‐stent method

**DOI:** 10.1111/1759-7714.15185

**Published:** 2023-12-27

**Authors:** Tadashi Sakaguchi, Maki Ohi, Yoichi Nishii, Osamu Hataji

**Affiliations:** ^1^ Matsusaka Municipal Hospital Respiratory Center Matsusaka Japan; ^2^ Matsusaka Municipal Hospital Radiology Department Matsusaka Japan

**Keywords:** AERO stent, anaplastic thyroid carcinoma, stent collapse, stent‐in‐stent

## Abstract

Tracheal AERO stent collapse is a rare complication compared to bronchial AERO stent collapse due to differences in the nitinol framework thickness. A 58‐year‐old man with a bulky anaplastic thyroid carcinoma was referred to our hospital due to exacerbation of tracheal stenosis despite the administration of lenvatinib. His tracheal stenosis exhibited a severe extrinsic compression pattern with a length of 8 cm. Because tracheotomy was inappropriate, we placed an 18 × 80 mm AERO stent. Five months later, he was readmitted with severe dyspnea due to collapse of the distal portion of the stent caused by tumor growth. Because stent removal was difficult, we placed an additional AERO stent (18 × 60 mm) to cover the collapsed portion. The additional stent successfully expanded the collapse and improved his dyspnea. To our knowledge, this is the first case where a tracheal AERO stent collapse due to a poor prognosis tumor was treated with the stent‐in‐stent method.

## Graphical abstract

A 58‐year‐old man with tracheal stenosis due to anaplastic thyroid carcinoma was treated with a tracheal AERO stent. He was readmitted with severe dyspnea due to collapse of the distal portion of the stent caused by tumor growth and remedied by stent‐in‐stent method. The stent‐in‐stent method with an additional stent was useful in managing tracheal AERO stent collapse, which is a rare complication compared to bronchial AERO stent collapse due to differences in the nitinol framework thickness.

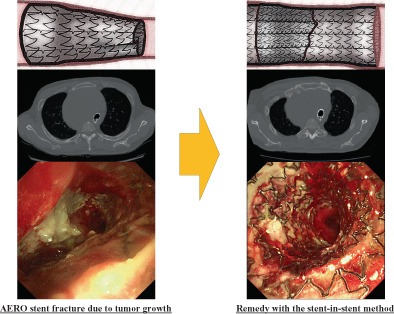

## INTRODUCTION

Central airway obstruction (CAO) caused by neck and chest tumors is a very dangerous oncological emergency with high mortality. When extrinsic compression is the main cause of obstruction, airway stent placement is the preferred method for palliation of dyspnea.^1^ AERO stents are relatively new, fully covered, self‐expandable metallic stents (SEMS), with strong radial force, approved in USA in 2007,^2^ and in Japan in 2014. The utility of AERO stents for airway stenosis in locally aggressive thyroid cancer cases remains unknown. Additionally, tracheal AERO stent collapse is a rare complication compared with bronchial AERO stent collapse, due to the nitinol framework of a tracheal AERO stent being thicker than that of a bronchial stent.^3^ Herein, we report a rare case which experienced a tracheal AERO stent collapse due to a bulky anaplastic thyroid carcinoma, remedied by stent‐in‐stent method.

## CASE REPORT

**FIGURE 1:**
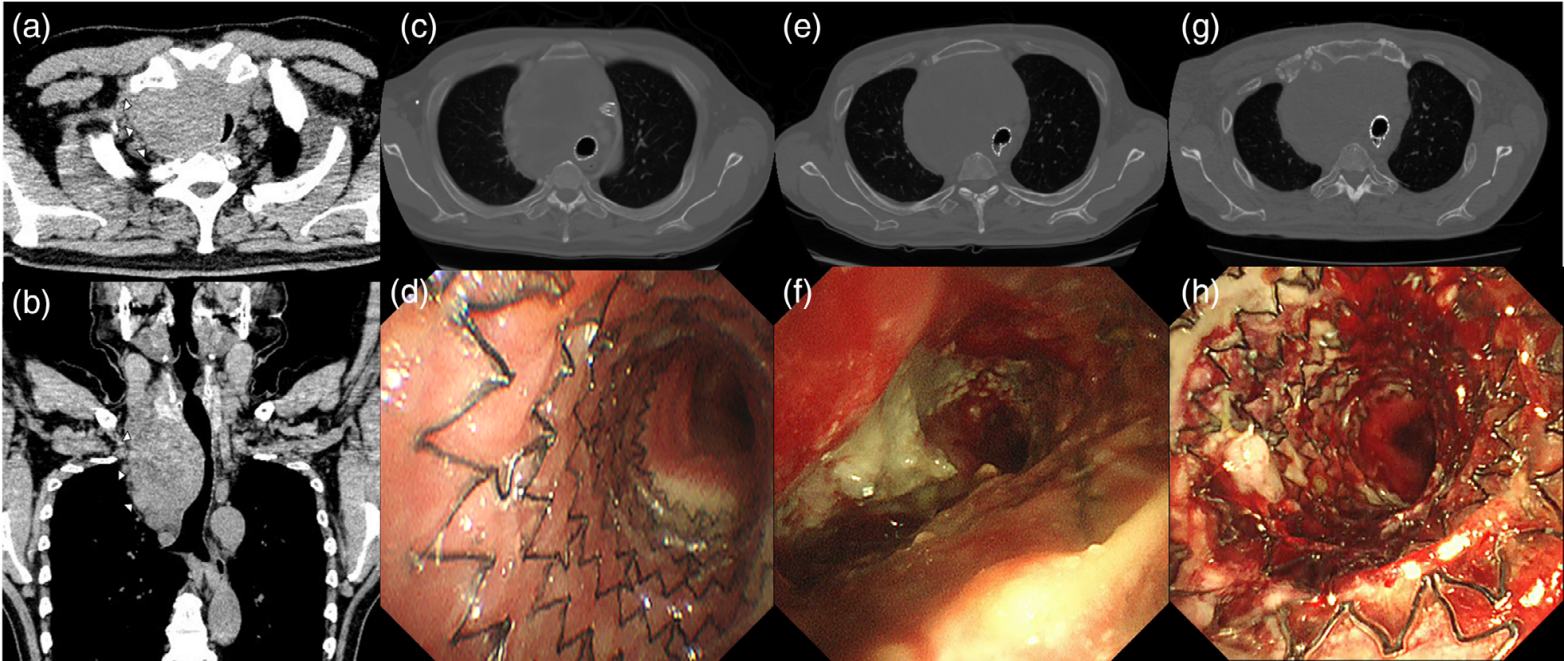
Computed tomography (CT) images of tracheal stenosis with severe extrinsic compression pattern (arrows) in cross‐sectional image (a), and in multiplanar reconstruction image (b). CT and bronchoscopic findings of the AERO stent distal portion; CT finding after first stent placement (c), bronchoscopic finding after first stent placement (d), CT finding at stent collapse by tumor growth (e), bronchoscopic finding at stent collapse by tumor growth (f), CT finding after additional stent placement with stent‐in‐stent method (g), bronchoscopic finding after additional stent placement with stent‐in‐stent method (h).

A 58‐year‐old man with throat discomfort and dysphagia due to a bulky mass in the anterior part of the trachea was diagnosed with anaplastic thyroid carcinoma (cT4bN0M0, stage IVB, UICC eighth edition) by the otolaryngologists. His medical history included hypertension and his blood tests showed no significant abnormalities. He was referred to our hospital due to exacerbation of tracheal stenosis despite administration of lenvatinib. His tracheal stenosis exhibited a severe extrinsic compression pattern with a stenosis length of 8 cm (Figure 1a,b). Tracheotomy was inappropriate because the tumor extensively invaded the anterior of his trachea. His airway was secured with a laryngeal mask airway under general anesthesia, and an 18 × 80 mm tracheal AERO stent (Merit Medical Systems) was placed by over the wire (OTW) delivery method under fluoroscopic guidance (Figure 1c,d). Five months later, he was readmitted with severe dyspnea due to collapse of the distal portion of the stent caused by tumor growth (Figure 1e,f). Stent removal was difficult because the proximal portion of the stent had caved in opposite the tracheal wall with granulation. His airway was secured with a rigid bronchoscope under general anesthesia, and an additional tracheal AERO stent (18 × 60 mm) was placed by OTW delivery method covering the collapsed portion (Figure 1g,h). The additional stent successfully expanded the collapsed portion of existing stent and improved his dyspnea.

## DISCUSSION

Anaplastic thyroid carcinoma, which is rare but carries a poor prognosis, leads to severe tracheal compression, airway compromise, and eventually death. It has been reported that tracheal stenting with Ultraflex‐covered stents is a relatively safe and effective method for palliation of distressing airway symptoms in patients with anaplastic thyroid carcinoma, although the survival benefit is minimal.^4^ The utility of the AERO stent for airway stenosis due to a locally aggressive anaplastic thyroid carcinoma remains unknown.

Malignant CAO can present as purely endoluminal tumors, extrinsic compression or a combination of both, and airway stent placement has the unique ability to quickly resolve extrinsic compressions.^5^

We considered tracheotomy to be inappropriate for this case because the tumor extensively invaded the anterior of the trachea, therefore an airway stent was appropriate. Among airway stents, silicone stenting would be unfavorable for the severe extrinsic compression of the trachea in this case because of the risk of migration, and difficulty in obtaining rapid airway security due to weak self‐expanding force and narrower internal diameter compared with SEMS. We therefore placed a tracheal AERO stent which is characterized as being self‐expandable, having technically easy placement, and sufficient radial force. In our case, the first placement of a tracheal AERO stent successfully improved the airway stenosis for 5 months without dyspnea. Severe dyspnea, however, occurred following stent collapse. One retrospective study reported that AERO stent collapse occurred in 5% of cases,^6^ all of which occurred with bronchial AERO stents due to the fragility of bronchial AERO stents resulting from thickness differences in the nitinol framework.^3^ When AERO stent collapse occurs, removal and replacement are considered.^6^ This procedure, however, is sometimes difficult due to excessive risk of airway perforation or massive hemorrhage. The usefulness of the stent‐in‐stent method with airway stents has been reported in the treatment of restenosis with granulation tissue hyperplasia and tumor growth,^7^, ^8^, ^9^, ^10^, ^11^ and the method would also be useful in dealing with stent collapse as in this case.

To our knowledge, this is the first case which experienced stent collapse of a tracheal AERO stent due to tumor progression, remedied by stent‐in‐stent method.

## AUTHOR CONTRIBUTIONS

**Tadashi Sakaguchi:** Conceptualization; methodology; resources; writing original draft. **Maki Ohi:** Writing – review & editing; supervision. **Yoichi Nishii:** Methodology; supervision. **Osamu Hataji:** Conceptualization; supervision.

## CONFLICT OF INTEREST STATEMENT

This case report did not receive any specific grants from funding agencies in the public, commercial, or not‐for‐profit sectors. Matsusaka Municipal Hospital, Respiratory Center received research grant funding from Novartis, GlaxoSmithKline, AstraZeneca, Daiichi Sankyo, Bayer, and Boehringer Ingelheim. O. Hataji received speaker fees as honoraria from Novartis Pharma, AstraZeneca, and Boehringer Ingelheim Japan. The remaining authors declare no conflict of interest.

## Supporting information


**VIDEO S1:** Bronchoscopic video data of stent collapse by tumor growth.

**VIDEO S2:** Bronchoscopic video data after additional stent placement with stent‐in‐stent method.
